# Tolerance of Chemoorganotrophic Bioleaching Microorganisms to Heavy Metal and Alkaline Stresses

**DOI:** 10.1155/2015/861874

**Published:** 2015-07-06

**Authors:** Annick Monballiu, Nele Cardon, Minh Tri Nguyen, Christel Cornelly, Boudewijn Meesschaert, Yi Wai Chiang

**Affiliations:** ^1^Laboratory for Microbial and Biochemical Technology (Lab *µ*BCT), Cluster for Bioengineering Technology, Department of Microbial and Molecular Systems, KU Leuven @ Brugge-Oostende (Kulab), 8400 Oostende, Belgium; ^2^Matériaux et Contrôles Physico-Chimiques, Département Mesures Physiques, Université de Bordeaux, 33175 Gradignan, France; ^3^School of Engineering, University of Guelph, Guelph, ON, Canada N1G 2W1

## Abstract

The bioleaching potential of the bacterium *Bacillus mucilaginosus* and the fungus *Aspergillus niger* towards industrial residues was investigated by assessing their response towards various heavy metals (including arsenic, cadmium, cobalt, chromium, nickel, lead, and zinc) and elevated pH. The plate diffusion method was performed for each metal to determine the toxicity effect. Liquid batch cultures were set up for more quantitative evaluation as well as for studying the influence of basicity. Growth curves were prepared using bacterial/fungal growth counting techniques such as plate counting, optical density measurement, and dry biomass determination. Cadmium, nickel, and arsenite had a negative influence on the growth of *B. mucilaginosus*, whereas *A. niger* was sensitive to cadmium and arsenate. However, it was shown that growth recovered when microorganisms cultured in the presence of these metals were inoculated onto metal-free medium. Based on the findings of the bacteriostatic/fungistatic effect of the metals and the adaptability of the microorganisms to fairly elevated pH values, it is concluded that both strains have potential applicability for further research concerning bioleaching of alkaline waste materials.

## 1. Introduction

The growing world population has resulted in an increase in materials consumption and industrial expansion, causing unsustainable pressures on the planet's natural resource reserves and the environment. For this reason, investigation concerning the development of sustainable technologies and recycling strategies is becoming favoured. In the last decades, interest in bioleaching, which is the conversion of solid metal values into their aqueous soluble forms using microorganisms, has increased significantly [1, 2]. This technology has been commercialized for some metal ores such as the biooxidation of refractory gold ores and in copper recovery [3, 4].

The solubilization of metals can be accomplished by various species of bacteria and fungi and is based on three main mechanisms that can occur simultaneously [2, 5, 6]. Acidolysis (i) is by far the most important one and is quite similar to the mechanism of conventional acid leaching. Organic and inorganic acids are produced by the microorganisms, which in turn leach the metals from the solids by protonation. In complexolysis (ii), biogenic agents are excreted from the microbes, and these solubilize metal ions through ligand formation. Citric acid, for instance, is a powerful natural chelating agent [7]. Lastly, in redoxolysis (iii), oxidation and reduction reactions occur that set metals free from the mineral.

Bioleaching is influenced by a wide range of parameters including physicochemical and microbiological factors. These affect both the growth of the microorganisms and their leaching behaviour. For bioleaching to be successful, it is obvious that optimal growth conditions must be maintained and the microorganism must be able to leach the material. In addition, and most importantly, the microorganism must be resistant to the metals that are leached out [1]. A suitable temperature, pH, oxygen, salinity, and the availability of nutrients are imperative for the growth and maintenance of microorganisms. These conditions are species dependent and it is principally important to know to which classification the microorganism belongs (autotrophic/heterotrophic, etc.). Although the optimal temperature mostly varies around 30–50°C (for mesophiles), bioleaching performed at lower/higher temperatures (psychro-/thermophiles) is also reported, as is the use of aerobic or anaerobic microorganisms [1, 8]. The pH of the medium is of great importance for the quantity and nature of the biogenic acids excreted and it also has an effect on the availability of heavy metals [9]. The production of organic acids is strongly dependent on the medium composition and the conditions of cultivation; in that respect, the carbon source and its concentration are important, as well as the presence of essential ions [10]. For the leaching of the metals, the properties of the solids including the mineralogical composition, slurry density, and particle size are key factors [5].

Besides the case for natural resources, bioleaching can be an interesting route for metal recovery from industrial waste materials, such as incinerator ashes, metallurgical slag, and mineral tailings. It can also be applied in the detoxification of soils and groundwater contaminated with heavy metals at various deposit sites [9, 11, 12]. However, challenges lie in the caustic and toxic nature of many of these waste materials. The presence of heavy metals in bioleached materials can adversely affect the growth and metabolic processes of microorganisms. Heavy metals are taken up by the bacterial cell through the same ion transport normally required for essential minerals such as magnesium and phosphate [13]. This leads to inhibition of specific enzyme activities, displacement of essential metals, and disruption of membrane transport processes [14, 15]. Bioleaching over a two-step process, in which there is no direct contact between the microorganisms and the released heavy metals, is preferable to reduce the toxicity effects; however, it complicates the implementation of continuous recovery systems [16].

There is no unequivocal answer on the tolerance of microorganisms to the presence of toxic heavy metal ions. It has been reported that some microorganisms acquire some form of adaptation mechanism, similar to what is seen upon exposure to antibiotics, to ensure the microbial population's survival and diversity. Metal tolerance mechanisms consist of either extracellular or intracellular sequestration of the toxic metal and are often gene-regulated by plasmids [17, 18]. Adaptation strategies include active transport of metal ions from the inside to the outside of the cell, interaction with extracellular polymeric substances (EPS), formation of cell surface complexes, and metal reduction to a less toxic state [9, 14, 19–21].

The best known tolerance strategy towards heavy metals among* Bacillus *species is the production of extracellular polymeric substances (EPS), a complex mixture of polysaccharides, proteins, nucleic acids, and lipids. The EPS production is influenced by the medium composition and is the bacterium's protection against environmental stress [22]. During the bioleaching process, it also contributes to the formation of biofilm, which plays an important role in the attachment of the bacterial cell to the solid material [19]. In the EPS layer, the optimal conditions for pH and solute concentration are maintained. Moreover, the EPS is involved in the capture of metal ions from the solution, preventing their entrance into the bacterial cell [23].

In the present study, it is investigated whether elevated pH and the presence of multiple heavy metals and metalloids hinder the feasibility of alkaline bioleaching of industrial residues. For this purpose, the tolerance of two chemoorganotrophic microbial strains that have shown potential for the bioleaching of waste materials [16],* Bacillus mucilaginosus* and* Aspergillus niger*, was assessed. These microorganisms are associated with silica-rich minerals in nature, compounds commonly found in alkaline industrial residues, but in their natural environment they are not exposed to, and thus do not become adapted to, high levels of mobile heavy metals and metalloids [24, 25].

## 2. Materials and Methods

### 2.1. Microbial Organisms and Chemicals

*Bacillus mucilaginosus* was obtained from China Center of Industrial Culture Collection (CICC, China).* Aspergillus niger* (DSM-872) was delivered by the Deutsche Sammlung von Mikroorganismen und Zellkulturen GmbH (DSMZ, Germany). As recommended by the suppliers,* B. mucilaginosus* was cultured on nutrient agar supplemented with 0.001% MnSO_4_·H_2_O for sporulation enhancement and incubated for 24 hours at 30°C, whereas* A. niger* was cultured on potato dextrose agar and incubated at 30°C for 5 days. Agar slants of each strain were preserved at 4°C.

The heavy metals {and respective salts} investigated were As(III) {KHAs_2_O_4_}, As(V) {KH_2_AsO_4_}, Cd(II) {Cd(NO_3_)_2_·4H_2_O}, Co(II) {Co(NO_3_)_2_·6H_2_O}, Cr(III) {Cr(NO_3_)_3_·9H_2_O}, Cr(VI) {K_2_Cr_2_O_7_}, Ni(II) {Ni(NO_3_)_2_·6H_2_O}, Pb(II) {Pb(NO_3_)_2_}, and Zn(II) {Zn(NO_3_)_2_·6H_2_O}. Each metal salt was dissolved in a citric acid/sodium citrate 100 mM buffer of pH 6.80. Stock solutions of 1000 ppm metal content were prepared, and further dilutions were made with the same buffer.

All growth media and chemicals were of analytical grade, purchased from Sigma Aldrich (Bornem, Belgium), and were used without any further purification. Growth media, chemical solutions, and glass materials were sterilized for 15 minutes at 120°C prior to use, and subsequent manipulations were executed under sterile conditions in a laminar flow cabinet.

### 2.2. Plate Diffusion Method

A first investigation on the effect of the aforementioned elements on the growth of the microbial strains was performed using the plate diffusion method; more specifically, the central well test was used [26]. In this method, a small cavity is made at the centre of an agar plate, into which an amount of metal solution is introduced. This allows the formation of a diffusion gradient over the agar plate during incubation, with higher concentration near the well and essentially nil concentration at the outer perimeter. A nutrient or potato dextrose agar plate (90 mm) was first abundantly inoculated with a freshly grown culture of, respectively,* B. mucilaginosus* or* A. niger* strain. Then, a central well of 1 cm was made. Into this well, 100 *μ*L of metal solution with a concentration of 1000 ppm of the metal component was added. Each plate was prepared in duplicate, and blank plates using buffer solution with no metal compounds were also made. After the desired incubation period at 30°C, the effect of the metals on the growth of the two strains was assessed visually by measuring and comparing the diameter of the growth inhibition zones. The plate diffusion test was subsequently repeated with lower concentrations of metal solution for those conditions in which sensitivity of the microbial strains occurred with the 1000 ppm solution, to assess the tolerance threshold.

### 2.3. Liquid Batch Cultures

The metal that demonstrated a significant impact on the growth of the two microbial strains during the aforementioned plate diffusion test was implemented in liquid cultures. In this test, a desired quantity of the metal stock solution was added to the inoculated broth medium (nutrient broth with 0.001% MnSO_4_·H_2_O for* B. mucilaginosus* and potato dextrose broth for* A. niger*). Reference blanks were prepared by adding an equal amount of buffer solution rather than metal solution to the broth medium.

For the experiments concerning the effect of basicity, the initial pH of the nutrient-rich medium (initial pH 7.15 for the nutrient broth and pH 6.70 for the potato dextrose broth) was increased with NaOH addition until the desired pH (9.0, 11.0, 12.0, and 13.0) was reached. The pH was measured with a SevenMulti pH meter (Mettler Toledo).

For each experiment, 200 mL or 100 mL of the broths, for the metal-stress and alkaline-stress experiments, respectively, was placed in Erlenmeyer flasks and inoculated with the appropriate strain. Incubation was performed at 30°C for the desired duration. The solutions were stirred by a magnetic bar. The experiments were performed in batch mode and were not pH-adjusted. The pH was measured every 24 hours.

### 2.4. Determination of Microbial Growth

Conventional techniques were used to detect the microbial growth over time. For* B. mucilaginosus*, the spread plate method and measurement of optical density were combined. For spread plating, a 100 *μ*L aliquot of the bacterial culture (whether or not serially diluted in physiological salt solution, 0.9% NaCl) was transferred to a nutrient agar plate and streaked across the entire surface of the plate. Every spread plate was made in duplicate. After incubation, colonies were manually counted and growth was expressed as colony forming units per millilitre (CFU/mL). Optical density was measured with a Jenway spectrophotometer at 610 nm wavelength. For this purpose, 1 mL samples of the culture broths were taken and, if necessary, diluted with nutrient broth so that the absorbance was in the linear range of 0–0.6. A zero-point calibration was performed with blank nutrient broth.

Growth of* A. niger* was followed by either the spread plate method or by the measurement of dried biomass. The spread plate method was analogous to the one for the* Bacillus* strain, except that potato dextrose agar plates were used. Measurement of dried biomass involved the fungal biomass being separated from the liquid culture with a Biofuge Stratos Centrifuge (Heraeus Instruments), followed by overnight drying at 110°C. The mass of the dried biomass is a measure for the growth of the fungal cells. These liquid batch cultures were set up with 400 mL of the broth medium. Additionally, the concentration of metal in the supernatant phase was determined and compared to the initial metal concentration. Metal concentration was determined using atomic absorption spectrometry (SpectrAA 220, Varian, Belgium).

## 3. Results and Discussion

### 3.1. Growth of* Bacillus mucilaginosus*

#### 3.1.1. Influence of Heavy Metals

**Figure 1:**
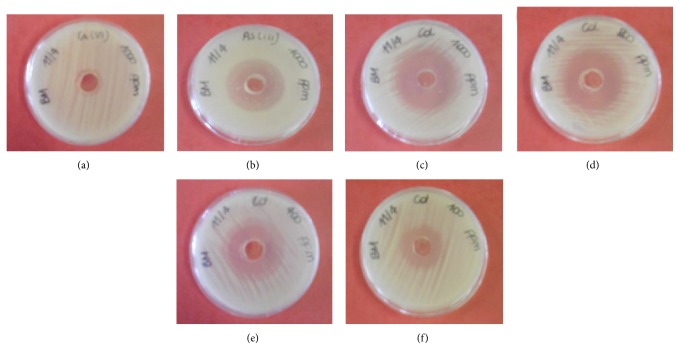
Visualization of the inhibition zones in the plate diffusion test for the investigation of the effect of (a) 1000 ppm (19.23 mM) Cr(VI), (b) 1000 ppm (13.35 mM) As(III), (c) 1000 ppm (8.90 mM) Cd(II), (d) 800 ppm (7.12 mM) Cd(II), (e) 400 ppm (3.56 mM) Cd(II), and (f) 100 ppm (0.89 mM) Cd(II) on the growth of* Bacillus mucilaginosus*.

The results of the plate diffusion test showed that the buffer solution has no influence on the growth of both* B. mucilaginosus* and* A. niger*. In Figure 1 the most important observations of heavy metal inhibition on* B. mucilaginosus* are shown, these being the presence of Cr(VI), As(III), and Cd(II). The other metals that were tested did not have a significant influence on the growth of* B. mucilaginosus* and are consequently not shown here. These tests were performed with 1000 ppm of metal compound; therefore, for each metal, the molar concentration differed. In the present case, the molar concentrations in descending order are 19.2 mM for Cr(VI), 13.3 mM for As(III), and 8.9 mM for Cd(II). As indicated by the size of the inhibition zones, the largest sensitivity occurred with Cd(II) (Figure 1(a)) followed by As(III) (Figure 1(b)); Cr(VI) had minimal effect (Figure 1(c)). Based on these findings, it is concluded that Cd(II) is the most toxic metal for the growth of* B. mucilaginosus*.

The plate diffusion test was repeated for Cd(II) with decreasing metal concentrations (800, 400, and 100 ppm); these results are shown in Figures 1(d) to 1(e). The decreasing sensitivity with respect to the decreasing concentration of the metal is clearly visible through the behaviour of the inhibition zones. Another interesting observation made was that older cultures seemed to be more sensitive to the heavy metal stress. Yet, over time, some of these colonies adapted and grew under the heavy metal stress. The age of the biomass culture can contribute to an altered and delayed adaptation strategy due to changes in cell size, wall composition, and extracellular product formation [9].

**Figure 2:**
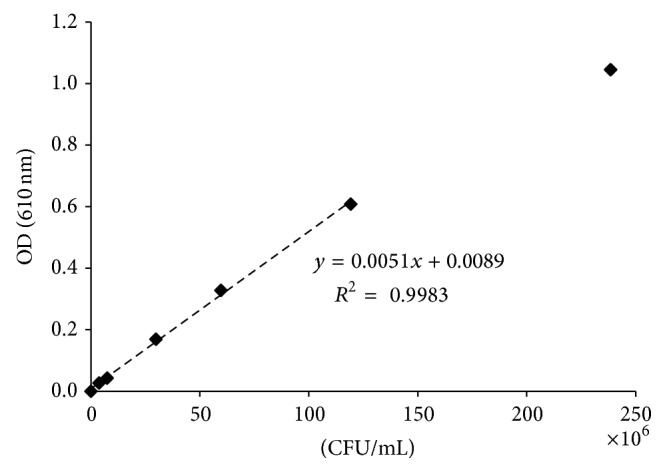
Correlation between cell count and optical density for* Bacillus mucilaginosus*.

Further investigation on the growth of* B. mucilaginosus* under heavy metal stress was performed in liquid batch cultures under the influence of Cd(II) and As(III) at 100 ppm concentration. In addition, 100 ppm of Ni(II) was also studied. The results were compared with a blank incubated batch where no metal was added to the inoculated nutrient-rich growth medium. Growth was measured over the incubation time in order to plot a bacterial growth curve.  Figure 2 demonstrates the correlation between cell count based on spread plating and the measurement of the optical density.* B. mucilaginosus *exhibits a linear gradient up to an optical density of about 0.6; higher densities of the* Bacillus* strain should thus be diluted. Measuring of the optical density has several advantages with respect to the plating of a culture [27]: it is of low cost and is rapid and nondestructive and therefore was preferred for the batch experiments performed with* B. mucilaginosus*.

**Figure 3:**
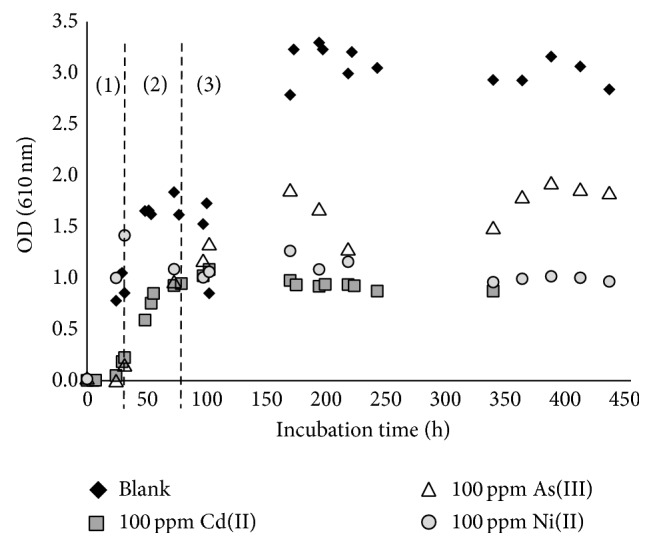
Growth curves of* Bacillus mucilaginosus* in broth in the presence of 100 ppm Cd(II) (0.9 mM), As(III) (1.3 mM), or Ni(II) (1.7 mM) compared to a blank medium without metal addition, determined according to optical density: (1) lag phase, (2) exponential phase, and (3) stationary phase.

In Figure 3, an overview of the different growth curves for* B. mucilaginosus* in the presence of the metals is given. Generally, it can be stated that a microbial growth curve consists of 4 phases as indicated in the figure: (1) the lag phase in which the microorganism is adapting to the new environment; (2) the exponential or logarithmic phase wherein the microorganisms grow and divide at a constant rate, the generation, or doubling time; (3) the stationary phase that indicates an equal growth and decay rate due to a lack of nutrients or an excess of waste materials produced by the microorganisms themselves; and finally (4) the death phase in which microorganisms die in an exponential way [28].

*B. mucilaginosus* grew under the influence of the metals; however, the lower optical density values obtained in the presence of metals reveal that the bacterial growth is much lower compared to the case where no metal is present. In comparison with the blank, growth in the presence of Cd(II) and Ni(II) was approximately one-third as large, while in the presence of As(III) approximately half of the maximum biomass concentration was reached. Taking into account the molar concentrations, once again Cd(II) (0.9 mM) is the most toxic metal over As(III) (1.3 mM) and Ni(II) (1.7 mM) during this test.

**Figure 4:**
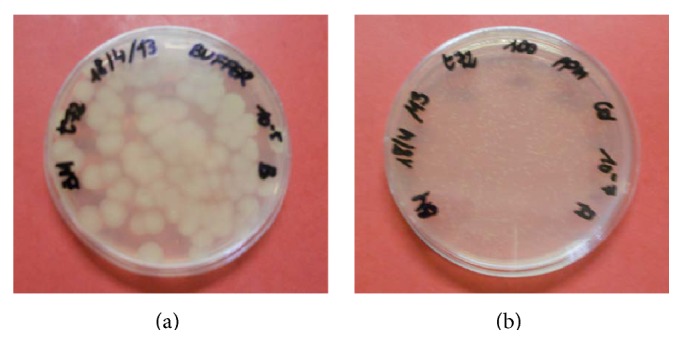
Growth of* Bacillus mucilaginosus* on nutrient agar plates. (a) Blank reference strain (after 72 hours of growth in batch culture, resulting in typical colony morphology on agar plate). (b) Strain that had previous contact with Cd(II) in a batch culture for 72 hours (note smaller colonies and the absence of mucous generation).

Each time the OD was measured, duplicate streak plates with metal-free agar medium were made for counting of the colonies. These tests showed that the number of CFU did not differ drastically for the cultures with and without metals, although the appearance of the colonies changed. Typically,* B. mucilaginosus* grows on the nutrient agar plates as large, smooth colonies with significant mucous generation (e.g., in Figure 4(a)). In the cases where the bacterial strain had contact with a heavy metal in the batch cultures, the colonies detected were much smaller, with apparently no mucous generation (e.g., in Figure 4(b)). It has been reported that, as part of the produced EPS, specific proteins can be formed that are capable of binding metal ions that promote the tolerance of the bacteria to heavy metals. The presence of such biomaterial can, however, also inhibit the microbial growth, for instance, through creating a delay in oxygen transfer or decreasing the removal of metabolic products from the bacterial cells [29]. The present results indicate that the surface or the surface complexes of* B. mucilagi*nosus were modified when the bacterium was initially grown in a medium with Cd(II) and subsequently was grown on a metal-free medium. It appears that the mucous metabolism ceased or was heavily disturbed.

**Figure 5:**
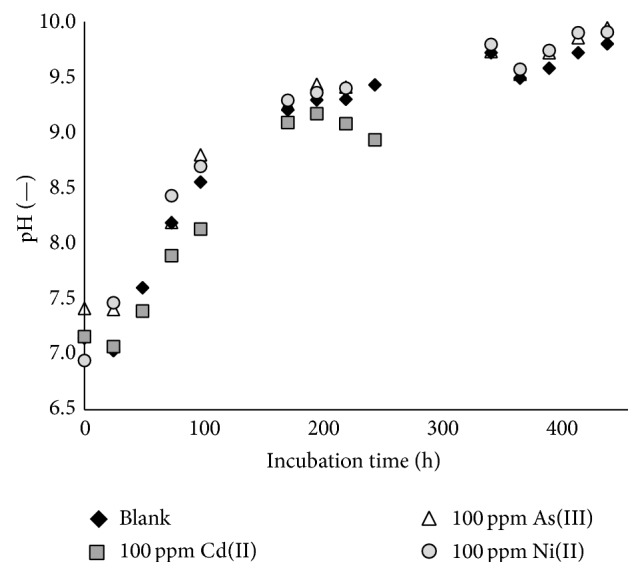
Variation of pH during growth of* Bacillus mucilaginosus* in a blank nutrient broth medium and under metal stress from 100 ppm Cd(II) (0.9 mM), As(III) (1.3 mM), or Ni(II) (1.7 mM) additions.

As shown in Figure 5, the pH during growth of* B. mucilaginosus* increased from 7 to 10 for both the blank culture and the cultures with metals. This can be explained by the citrate metabolism of* B. mucilaginosus* where citric acid, available from the buffer solution in which the metal salts were dissolved, is utilized as a carbon source, resulting in the alkalinization of the medium. It thus appears that this citric acid metabolism is not influenced by the presence of metals. It should be mentioned that, at such high pH, the metals can precipitate in their hydroxide forms [30]. This may explain why the bacteria do not become entirely inactivated, as the metal may no longer be bioavailable in solution.

#### 3.1.2. Influence of Initial pH

**Figure 6:**
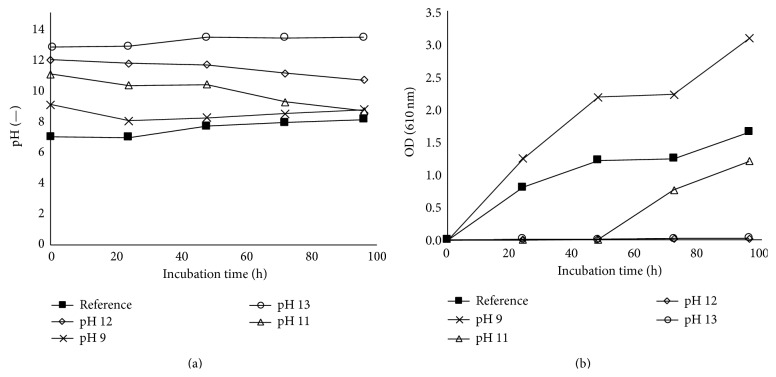
Evolution of pH (a) and OD (b) during the growth of* Bacillus mucilaginosus *in nutrient broth medium with different initial pH values.

In addition to the investigation of the impact of heavy metals, the influence of the initial pH value was examined. Metal-rich waste materials such as metallurgical slag and incineration ashes are typically characterized by their high basicity, and for this reason the* Bacillus* strain was cultivated under the initial pH values of 9, 11, 12, and 13.  Figure 6 shows the evolution of the pH and the growth of the* Bacillus* strain over the incubation time. Based on the optical density measurement, there is more growth of* B. mucilaginosus* when the pH of the nutrient broth was increased to 9 in comparison with the reference nutrient broth medium with a pH of 7. There was hardly any growth observed when the initial pH of the medium was increased to 12 and 13. For the culture with the initial pH of 11, exponential growth was observed starting from day 3; notably, at that moment the pH had decreased to about 9. These results are confirmed by the measurement of the weight of dried biomass at the end of these experiments (Table 1). No biomass was recovered for the cultures with the initial pH values of 12 and 13, while 0.88 g/L of biomass was found for the culture with initial pH of 9 and 0.26 g/L biomass for the one with initial pH of 11. Therefore growth of* B. mucilaginosus* was favoured at an initial pH of 9, whereas growth was delayed at the initial pH of 11; it appears that the bacteria secrete acidic compounds to adjust the pH to its best growth condition. An initial pH of 12 or 13 resulted in complete inactivation of the bacteria; in this case, the bacteria died off before being able to sufficiently acidify the medium.

### 3.2. Growth of* Aspergillus niger*

#### 3.2.1. Influence of Heavy Metals

**Figure 7:**
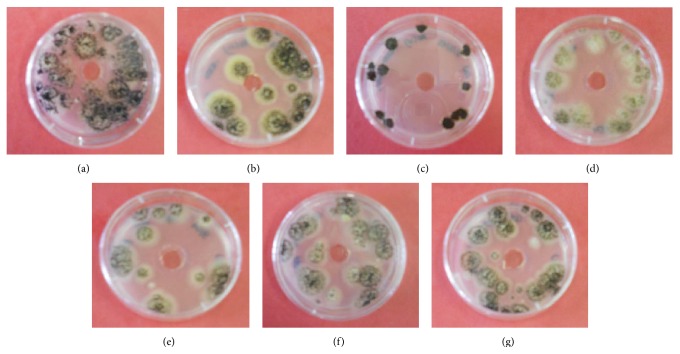
Visualization of the inhibition zones in the plate diffusion test for the investigation of the effect of (a) buffer solution, (b) 1000 ppm (13.35 mM) As(III), (c) 1000 ppm (13.35 mM) As(V), (d) 1000 ppm (8.90 mM) Cd(II), (e) 600 ppm (5.34 mM) Cd(II), (f) 400 ppm (3.56 mM) Cd(II), and (g) 100 ppm (0.89 mM) Cd(II) on the growth of* Aspergillus niger*.

The same experiments were performed on the fungal species* Aspergillus niger*. The most significant heavy metals that inhibited* A. niger* were As(III), As(V), and Cd(II). Several studies have reported the high tolerance of* A. niger* to heavy metals in comparison with other fungi, with Cd(II) being one of the most toxic metals for* Aspergillus *sp. [20, 31]. From Figure 7, it can be seen that the growth patterns of* A. niger* are different from that of* B. mucilaginosus*; as a reference, a plate without metal addition is shown in Figure 7(a). It is clear that As(V) and Cd(II) have a negative impact on the growth of the fungi. The test was repeated for Cd(II) with decreasing concentrations of 600, 400, and 100 ppm. These results are shown in Figures 7(e) to 7(g), in which significantly greater growth is seen for the lower concentrations.

**Figure 8:**
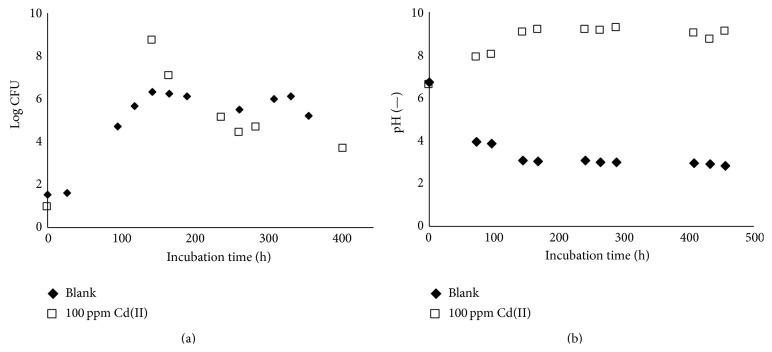
Growth of* Aspergillus niger* determined by spread plate colony counting (a) and evolution of pH (b) over incubation time in blank potato dextrose broth medium and in the presence of 100 ppm Cd(II) (0.9 mM).

**Figure 9:**
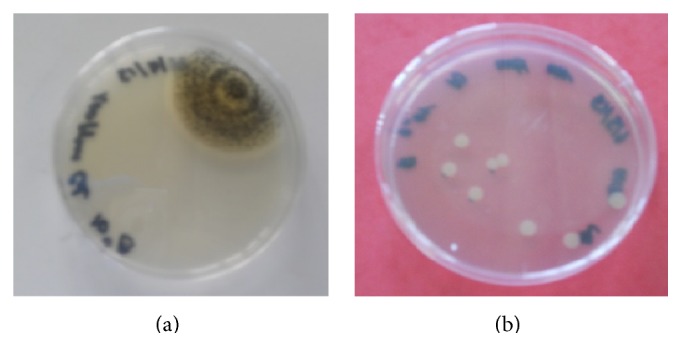
Comparison of growth of* Aspergillus niger* inoculated on potato dextrose agar plates after batch incubation of 10 days in (a) blank medium (normal growth) and (b) medium containing 100 ppm Cd(II) (0.9 mM).

A liquid batch culture was prepared for* A. niger* with 100 ppm of Cd(II). Just as in the case for* B. mucilaginosus*, fungal growth and pH were measured over the incubation time and compared to a metal-free reference culture. These results are provided in Figure 8, in which the fungal growth is calculated on the basis of the number of colony forming units. The results are similar to that of* B. mucilaginosus*; there is no significant difference in the CFU values, but the colony structure clearly changed. While* A. niger* usually grows as large, rough colonies with visible black spores, in the case of the strain that had contact with Cd(II), the colonies were appreciably smaller and lacking visible sporulation. This visualization is shown in Figure 9. Such results suggest that the metals are bound to substances on the cell surface as complexes, which dissociate once growth conditions return to optimal.

Figure 8 shows that the pH of the reference culture eventually dropped below 3, while for the culture exposed to Cd(II) the pH increased up to nearly 10.* A. niger* is known to produce organic acids during its metabolic activity, such as citric acid and gluconic acid [12]. The pH increase of the Cd(II) doped culture does not necessarily mean that there was no acid production, but it possibly means that basic compounds were produced in greater quantity. However, previous work performed on* Penicillium chrysogenum* [32] points to the disruption of the acid production metabolism being the primary cause, as the glucose oxidase activity is severely affected by heavy metals. This enzyme catalyzes the oxidation from glucose to gluconic acid. The reduction or absence of gluconic acid production could thus explain the pH increase.

An additional experiment using* A. niger* was performed to determine the dry weight of the produced biomass. The fungi were grown in 100 ppm Cd(II) broth and compared against a reference culture. After 15 days of growth, the fungal biomass was harvested. The reference culture contained 3.02 g/L dried biomass, while the culture grown in Cd(II) presence only amounted to 0.96 g/L of dried biomass. These results further support the conclusion that Cd(II) negatively influences the growth of* A. niger*. Notably, at the end of this experiment, the Cd(II) concentration decreased from the initial 100 ppm to roughly 80 ppm, indicating sorptive behaviour of the biomass to this metal. The biosorption capacity of filamentous fungi, such as* Aspergillus *sp. and* Penicillium *sp., has been demonstrated and is effective with both living and dead biomass material [33]. Fungal cell walls are complex structures consisting of chitins, proteins, polysaccharides, and other derivatives, providing many functional groups that are able to bind metal ions. This has even given rise to technological approaches where the biomass is altered to yield higher bioadsorption [9].

#### 3.2.2. Influence of Initial pH

**Figure 10:**
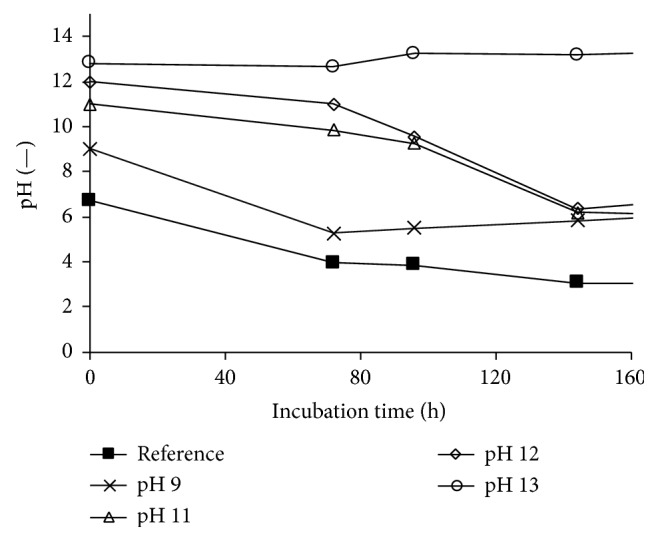
Evolution of pH during the growth of* Aspergillus niger *in potato dextrose broth medium with different initial pH values.

For the investigation of higher initial pH values, the* Aspergillus* strain was grown in potato dextrose broths with initial pH values of 9, 11, 12, and 13, and growth was compared with the reference culture at pH 6.7. As can be seen in Figure 10, which shows the evolution of the pH during the incubation, the pH decreased for the cultures prepared in initial pH lower than or equal to 12. In the culture with the initial pH of 13, the pH remained unchanged, which indicates the absence of microbial activity. Still, the pH of the reference culture decreased to the lowest value. At the end of the experiments, the dry weight of the biomasses was determined (Table 1): for the culture with initial pH of 9, 0.55 g/L of biomass was measured, while the biomass concentrations for the cultures with initial pH values of 11 and 12 were 1.23 g/L and 1.36 g/L, respectively. Hardly any growth was seen for the culture at pH 13 (mass not measured). It has been reported that* A. niger* presents advantages for bioleaching of incinerator fly ash because of its ability to thrive at higher basicity. In such case, the conditions reportedly become more favourable for gluconic acid production, which becomes the main bioleaching agent over citric acid [12]. From the present results it also appears that biomass growth is promoted at higher pH, as long as it does not exceed a certain limit.

## 4. Conclusions

The purpose of this study was to evaluate whether heavy metals and high basicity values hinder the growth of two chemoorganotrophic microorganisms, the bacterium* Bacillus mucilaginosus* and the fungus* Aspergillus niger*. Growth was determined in agar plates and in liquid batch cultures, both provided with the essential nutrients. The results showed that cadmium, nickel, and arsenite (As(III)) had a negative influence on the growth of* B. mucilaginosus*, whereas* A. niger* was sensitive to cadmium and arsenate (As(V)). These influences were characterized by delayed or reduced growth, but in none of the batch cultures a total inactivation or dying off of the microorganisms was observed for the 100 ppm concentration being used. Both microorganisms exhibited strong adaptation abilities, and growth was restored once the metal was removed. However, the colony appearance changed; therefore it seems that the metals cause modifications to exterior cell structures. Moreover, with the* Aspergillus* strain, a modification of the metabolism was detected. The incubation at higher pH values proved that both microorganisms are able to grow at higher basicity, up to pH 9 for* B. mucilaginosus* and even pH 12 for* A. niger*, values that are reasonably within the range of bioleaching of alkaline materials.

In conclusion, the investigated metals had a bacteriostatic and fungistatic effect on* Bacillus mucilaginosus* and* Aspergillus niger*, respectively, but did not act as bactericides or fungicides. With respect to the bioleaching of industrial residues, growth at elevated pH should not be an obstacle. Hence both microorganisms herein studied provide opportunities for application in the remediation of heavy metal laden waste materials, soils, or sediments.

## Figures and Tables

**Table 1:** Biomass dry weight concentration (g/L) after 96 hours as a function of initial pH during the growth of *Bacillus mucilaginosus* in nutrient broth medium and of *Aspergillus niger* in potato dextrose broth medium.

pH	*Bacillus mucilaginosus *	*Aspergillus niger *
9.0	0.88	0.55
11.0	0.26	1.23
12.0	No growth	1.36
13.0	No growth	Negligible
